# Simple Mistakes Causing Catastrophic Complications: Central Venous Catheter Removal Leading to Cerebral Air Embolism

**DOI:** 10.1155/crcc/8590063

**Published:** 2025-09-16

**Authors:** Sumair Ozair, Ishwor Sharma, Hafiz Ali Muhammad Raza, Sushil Khanal, Renee Walters, Bradley Boldizar

**Affiliations:** ^1^ Department of Internal Medicine, North Mississippi Medical Center, Tupelo, Mississippi, USA

## Abstract

Central venous catheters (CVCs) are commonly placed in patients in critical care units (CCUs) for a variety of reasons. Indications for CVC placement include rapid volume resuscitation, central venous pressure monitoring, venous access in patients with severe vascular disease, hemodialysis, and the need for the administration of vasoactive/bioactive medications. The placement of a CVC, however, does not come without risks to the patient, and one must keep these complications in mind. A 66‐year‐old male with Stage IV chronic kidney disease was admitted for dehydration secondary to diarrhea and was started on intravenous fluid resuscitation. During his eventful hospital course, the patient was transferred to the CCU, where a right internal jugular CVC was placed with eventual removal. A few minutes after removal, the patient was found to be poorly responsive, diaphoretic, and noted to have neurologic findings. A computed tomography scan of the head and a computed tomography angiogram of the head and neck revealed air within the subarachnoid space, subtle parenchymal hypodensity along the right cerebral cortex, and air inside the jugular and vertebral venous system. Magnetic resonance imaging of the brain revealed air within the cavernous sinuses, cortical veins, and dural sinuses. The patient was treated with aspirin and statin therapy given stroke‐like symptoms, with eventual improvement and discharge. Air embolism (AE) is an uncommon and dangerous complication that can result from various reasons, such as trauma, surgery, septal defects, or barotrauma. In this case, the AE was a devastating complication of a CVC. They can occur at various portions of the insertion and removal process. AE may cause cardiopulmonary distress and/or neurologic symptoms. Given the clinical context, a high level of suspicion is required to diagnose cerebral AE. This unfortunate event highlights the dangerous complications of a routine procedure. Early diagnosis and clinical suspicion of AE decrease morbidity and mortality.

## 1. Introduction

Air embolism related to the insertion or removal of a central venous catheter (CVC) is a rare but life‐threatening complication that may even lead to death. It can be either venous or arterial. Venous air embolisms occur because of the entry of air into systemic veins, whereas arterial air embolisms result from air entering pulmonary veins or directly into systemic arteries [1]. A study on the long‐term prognosis of patients admitted to a referral academic center with iatrogenic gas embolism revealed a crude mortality rate of 21% [2]. Additionally, neurological sequelae were noted in half of the survivors [2].

CVCs are commonly placed on patients in critical care units for a variety of reasons. Some important indications for CVC placement include rapid volume resuscitation, central venous pressure monitoring, venous access in patients with severe vascular disease, hemodialysis, and the administration of vasoactive or bioactive medications [3, 4]. The placement of a CVC does not come without risks to the patient, and one must keep these complications in mind when deciding to place a CVC. We describe a case of cerebral air embolism resulting from the removal of a CVC. Moreover, we discuss ways to prevent air embolism related to CVCs.

## 2. Case Presentation

A 66‐year‐old male with a past medical history of hypertension, heart failure with a reduced ejection fraction, coronary artery disease, and Stage IV chronic kidney disease presented to the hospital due to 1 week of diarrhea with abnormal laboratory findings identified during a nephrology clinic visit. The laboratory findings were consistent with worsening kidney dysfunction, which was likely due to volume loss in the setting of his week‐long bout of diarrhea. He was started on intravenous fluid resuscitation at the time of admission.

During his hospitalization, he suddenly became bradycardic, hypoxic, and hypotensive. The patient was found to have pulmonary edema, and he was ultimately transferred to the intensive care unit (ICU), where he required intubation and vasopressors. Given his current clinical status with the use of vasoactive medications, a right internal jugular CVC was placed. Additionally, he was noted to have worsening kidney function, requiring hemodialysis via a left forearm arteriovenous fistula. This episode of pulmonary edema and worsening kidney function was attributed to the administration of intravenous fluids coupled with his reduced cardiac function, resulting in acute heart failure exacerbation.

As he clinically improved, he was weaned off the ventilator and pressure support, resulting in extubation, and was subsequently transferred out of the ICU. Since he no longer required vasoactive medications, an order was placed for the removal of his right internal jugular CVC. As the central line was removed, the patient was placed in the reverse Trendelenburg position, which did not align with the protocol set by the hospital and the universally accepted protocol. Immediately after the removal of the central line, a sterilized gauze dressing was placed with external pressure applied for 5 min. At the time of removal, the patient was alert and oriented. After 5 min of external pressure, the dressing was taped in place, and the patient remained in a seated position.

Several minutes later, he was found to be poorly responsive and diaphoretic. He was found to be euglycemic and normotensive, with an oxygen saturation of 97% on room air; however, he was tachycardic. Additionally, on physical examination, the patient had a left lateral gaze preference for which a stroke alert was activated and neurology consulted. A computed tomography (CT) scan of the head and a CT angiography (CTA) image of the head and neck were obtained immediately. While awaiting imaging results, the patient experienced focal seizures, which were treated with benzodiazepines. Given the lack of airway protection, the patient was transferred to the ICU and was emergently intubated and started on antiepileptic drugs. EEG was ordered for further evaluation.

**Figure 1:**
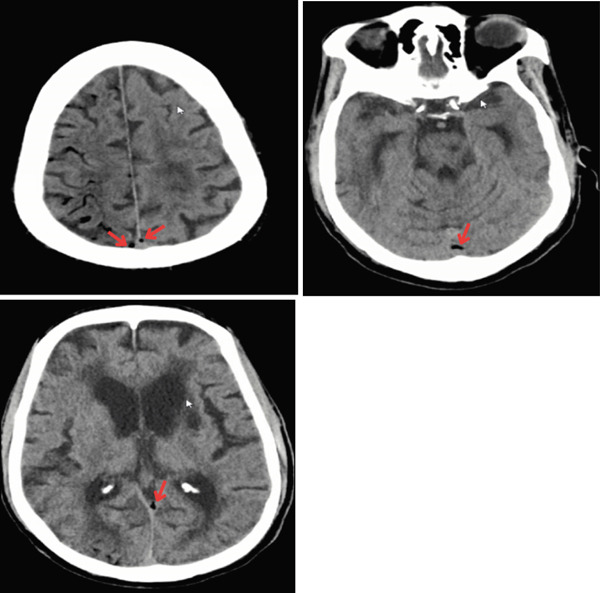
CT scan of the brain showing air embolism in the cerebral venous system, marked by red arrows.

**Figure 2:**
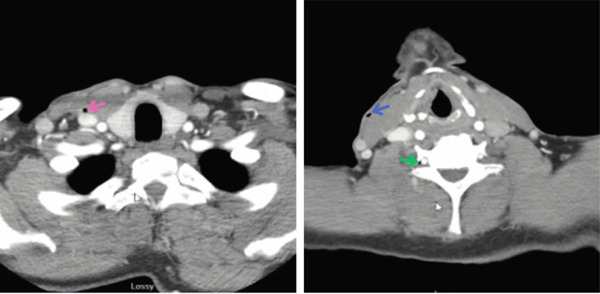
A CTA scan of the head and neck revealed air within the jugular venous system, as indicated by the pink arrow. A CTA scan of the head and neck revealed air in the track created by the central venous catheter under the skin (indicated by the blue arrow) and air in the vertebral venous system (indicated by the green arrow).

**Figure 3:**
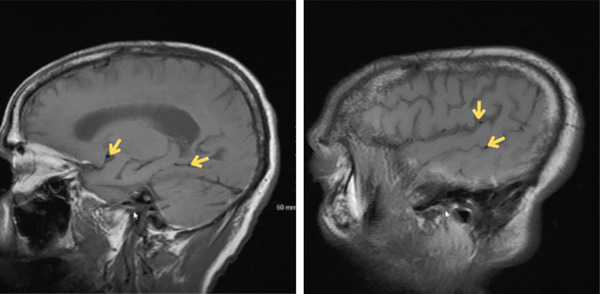
MRI of the brain showing air in the dural venous sinuses, highlighted by yellow arrows. MRI of the brain revealed air within the cerebral veins, as highlighted by yellow arrows.

CT of the head (Figure 1) revealed air within the subarachnoid spaces along the right cerebral cortex and subtle parenchymal hypodensity along the right cerebral cortex. CTA of the head and neck revealed 56% stenosis of the proximal left cervical internal carotid artery and moderate stenosis of the bilateral intracranial internal carotid arteries. CTA also revealed air inside the jugular and vertebral venous system (Figure 2). The patient did not exhibit evidence of large vessel occlusion. An electroencephalogram revealed numerous brief focal epileptiform discharges. Further imaging was conducted with magnetic resonance imaging (MRI) of the brain (Figures 3), revealing air within the cortical veins and dural sinuses with no arterial air embolism.

He was given full‐dose aspirin and started on high‐intensity statin due to clinical signs resembling stroke. Hyperbaric oxygen, although available, could not be immediately initiated because of the unavailability of in‐house staff who were trained in its administration at that time. Again, the patient clinically improved, resulting in another extubation, and was transferred to the medical floor once more. Nine days later, the patient was discharged to a nursing home with minimal neurological deficits.

## 3. Discussion

Air embolism is an uncommon and potentially dangerous complication that can occur for various reasons, with incidence rates ranging from 0.01% to 2% [5, 6]. They can be either venous or arterial and may result from intravenous catheter insertion or removal, trauma, surgery, septal defects, or barotrauma from positive pressure ventilation [7, 8].

Patients with air embolisms may develop sudden‐onset respiratory distress, substernal chest pain, and/or neurological symptoms such as lightheadedness or dizziness. They may even be life‐threatening, causing hemodynamic collapse and cardiac arrest [9].

Venous air embolisms have been documented to occur during neurosurgical or otolaryngological interventions due to low pressure in the dural venous system, with patients sitting up during these types of procedures [4, 7, 10]. Similarly, barotrauma, resulting either from positive pressure ventilation or rapid ascent in scuba diving, often leads to a loss of pulmonary vascular integrity, which in turn causes air embolism due to alveolar rupture and subsequent air entry into the pulmonary circulation [4, 11].

Air embolism may also be a devastating complication of CVC, as demonstrated in the case above. Air embolism can occur during the insertion or removal or may even occur at any time a patient has a CVC. During insertion, an air embolism may occur if there is a failure to occlude the needle base or if the catheter lumen is not capped. This risk is heightened if the patient is placed in an upright position or is taking deep inspiration. Additionally, air embolisms can result from accidental disconnections of the catheter or from fractures of the catheter itself [4, 12]. During the removal of a CVC, air embolism may result from decreased central venous pressure, especially when the CVC is positioned in the reverse Trendelenburg position or is deeply inspired [4], and a persistent skin tract leading to the vasculature from the longstanding CVC [13]. Table 1 shows the measures that should be taken to prevent air embolism during CVC insertion.

**Table 1:** Measures that should be taken to prevent air embolism during CVC insertion, maintenance, and removal.

**During insertion and maintenance**	**During removal**
Securely close off the needle hub and catheter	Place patient in head‐down position (Trendelenburg) unless contraindicated
Ensure all connections tight	Instruct the patient to perform the valsalva maneuver or a full inspiration with breath holding, followed by quick CVC removal
Secure all unused hubs with locks	Apply positive end‐expiratory pressure to patients on mechanical ventilation
Inspect the catheter and all connections for integrity; look for any cracks or broken seals	As soon as the CVC is removed, apply a vaseline occlusive dressing
Ensure that air is fully expelled from syringes and they are primed before connecting to the catheter	Apply pressure to the removal site immediately after CVC removal for a minimum of 10 min
Exercise caution during patient transfers or movement to avoid accidental pulling of the catheter	Monitor the patient for any signs of bleeding for at least 30 min post‐CVC removal

*Note:* Modified from Brockmeyer et al. [4] and Wong et al. [14], used with permission.

Abbreviation: CVC, central venous catheter.

Typically, pulmonary air embolism is the result of an air embolism that is located in the venous system. Similarly, the retrograde flow of air in the jugular venous system leads to cerebral air embolism [3]. In the case of septal defects or patent foramen ovale, venous air embolism may paradoxically embolize the arterial system [3]. It is also possible that air in the venous system passes through the pulmonary capillaries to reach the arterial side and causes end‐organ damage given that the air burden is significant [15].

The clinical effects of air embolism largely depend upon the volume of air and the rate at which it is introduced into the circulatory system. A minimal air embolism is believed to be asymptomatic or minimally symptomatic, whereas a large volume may lead to hemodynamic instability and can be fatal [16]. In the appropriate clinical context, a high level of suspicion is required to diagnose cerebral air embolism [10]. Neurologic symptoms during the insertion or removal of CVCs should trigger suspicion of air embolism even in the absence of intracardiac shunts [17].

The management of air embolisms includes airway, breathing, and circulatory management. Other supportive therapies include oxygen, mechanical ventilation, volume resuscitation, and vasopressors if clinically indicated. In cases of cardiopulmonary arrest, an advanced cardiovascular life support protocol should be initiated. The patient should be positioned in the left lateral decubitus position with the head down (Durant’s maneuver) for venous embolism and supine for arterial embolism. The advantage of Durant’s maneuver is that it keeps any air trapped within the heart away from exiting the right ventricle, thereby reducing the blockage of the pulmonary vasculature by an air bubble [3, 8]. Hyperbaric oxygen therapy is indicated whenever there are neurologic deficits as a result of air embolism. Hyperbaric oxygen allows the creation of high gradients for nitrogen to be displaced from inside the air bubble, reducing the size of the air bubble in addition to increasing the oxygen tension at tissue and reducing tissue ischemia [18]. Patients who have generalized seizures should be treated with an appropriate antiepileptic drug [19]. Prevention of air embolism is key, and all efforts should be made to prevent air embolism during the insertion or removal of the catheter (Table 1). Diagnostic tests include arterial blood gas analysis, echocardiogram with bubble study, chest radiography, and CT or MRI of the brain. A delay in imaging may yield negative imaging results as the air becomes absorbed with time [14].

Following cerebral air embolism in our patient, a safe solution system was activated. After an appropriate investigation of the event, our facility made policy changes for the removal of CVCs (Table 2). This included creating a checklist for catheter removal and providing regular training to health care workers on proper CVC removal techniques, as well as ensuring strict adherence to the protocol or checklist.

**Table 2:** Central venous catheter removal policy.

1. Order for removal should be verified
2. Procedure should be explained to patient
3. Patient should be placed in supine flat or Trendelenburg unless contraindicated. (Trendelenburg position is contraindicated in clients with head injuries, intracranial pressure, and spinal cord injuries)
4. General condition of device and site should be inspected
5. If a PICC line, circumference of upper arm should be measured
6. All infusions into device should be discontinued
7. Perform hand hygiene, apply PPE, remove dressing, observe site for any redness, drainage, swelling, or pain. If observe site for any redness, drainage, swelling, or pain. If any of the above noted, obtain an order for a culture of the catheter tip
8. Remove gloves, perform hand hygiene, and put on sterile gloves. Remove sutures as needed, maintaining surgical asepsis
9. Using nondominant hand, apply sterile 4 × 4 − *i* *n* *c* *h* gauze to site. Instruct patient to perform Valsalva maneuver. If this is not possible or patient is on ventilator, remove at end of expiration
10. Grasp hub of device, slowly remove catheter in smooth, continuous motion an inch at a time. (If resistance met, catheter should be taped in place and physician should be notified) A. Constant pressure should be applied to exit site until bleeding stops. Then, apply sterile occlusive dressing (Vaseline gauze), and monitor as necessary B. Inspect device for appropriate length and for signs of jagged, uneven edges suggestive of breakage. Measure length if documented at time of insertion for comparison
11. Protective equipment should be removed. Perform hand hygiene
12. Encourage patient to lie flat for 30 min
13. Document

## 4. Conclusion

A high index of suspicion is necessary to diagnose cerebral air embolism in the right clinical context to detect this exceptionally rare complication. Our case report highlights the importance of adhering to established protocols for central line insertion and removal to prevent poor outcomes. Physicians should be well prepared to address this complication. Prevention is key, and early diagnosis can help decrease morbidity and mortality. Our goal is to raise awareness of this life‐threatening complication from CVC removal with the hope of early identification for expedient intervention.

## Consent

No written consent has been obtained from the patient as there is no identifiable data included in this case report.

## Conflicts of Interest

The authors declare no conflicts of interest.

## Funding

No funding was received for this manuscript.

## Data Availability

The data that support the findings of this study are available from the corresponding author on reasonable request.
